# Bariatric Surgery: Targeting pancreatic β cells to treat type II diabetes

**DOI:** 10.3389/fendo.2023.1031610

**Published:** 2023-02-15

**Authors:** Tiantong Liu, Xi Zou, Rexiati Ruze, Qiang Xu

**Affiliations:** ^1^ Department of General Surgery, Peking Union Medical College Hospital, Beijing, China; ^2^ School of Medicine, Tsinghua University, Beijing, China; ^3^ Chinese Academy of Medical Sciences and Peking Union Medical College, Beijing, China

**Keywords:** bariatric surgery, type II diabetes mellitus, β-cell, islet regeneration, vertical sleeve gastrectomy, Roux-en-Y gastric bypass

## Abstract

Pancreatic β-cell function impairment and insulin resistance are central to the development of obesity-related type 2 diabetes mellitus (T2DM). Bariatric surgery (BS) is a practical treatment approach to treat morbid obesity and achieve lasting T2DM remission. Traditionally, sustained postoperative glycemic control was considered a direct result of decreased nutrient intake and weight loss. However, mounting evidence in recent years implicated a weight-independent mechanism that involves pancreatic islet reconstruction and improved β-cell function. In this article, we summarize the role of β-cell in the pathogenesis of T2DM, review recent research progress focusing on the impact of Roux-en-Y gastric bypass (RYGB) and vertical sleeve gastrectomy (VSG) on pancreatic β-cell pathophysiology, and finally discuss therapeutics that have the potential to assist in the treatment effect of surgery and prevent T2D relapse.

## Introduction

Entering the 21^st^ century, the prevalence of obesity is increasing and posting a significant public health threat to most countries worldwide (1). Traditional treatment options for obesity include diet control, physical exercise, and pharmacological interventions (2). However, severe obesity is difficult to treat. It is widely recognized that pharmacotherapy and lifestyle intervention alone will barely lead to weight loss in most cases, while bariatric surgery (BS) is the most effective and sustainable strategy to achieve short- and long-term weight loss in such a setting (3).

Over the past few decades, the number of bariatric surgeries performed has increased exponentially worldwide for good reasons (4, 5). Studies have demonstrated that the BS contributes to sustained weight loss and facilitates the management of obesity-associated comorbidities, including cardiovascular, respiratory, musculoskeletal, reproductive, and renal diseases, and also experiences significant improvement (6–10). The general mental health status and patient-reported life quality also significantly increased after the surgery (3, 11–14). For another, the popularity of minimally invasive surgery and the increased number of qualified surgeons and surgical centers that perform BS has revolutionized the procedure by vastly increasing its safety profile. Moreover, from a public health perspective, the prevalence of BS can efficiently reduce the healthcare burden upon individuals and cut the overall healthcare cost (15).

**Figure 1:**
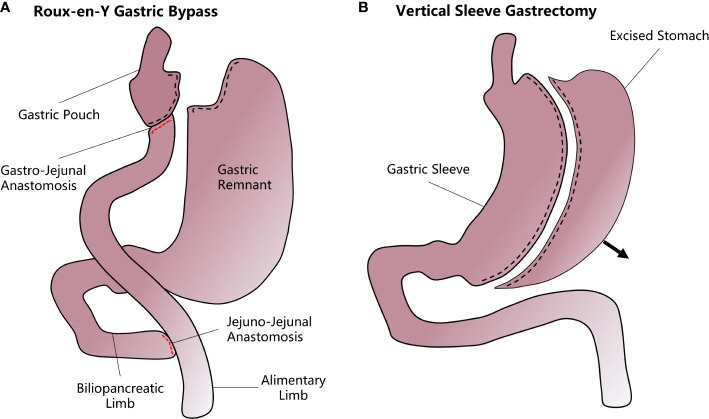
A diagram of how vertical sleeve gastrectomy (VSG) and Roux-en-Y gastric bypass (RYGB) are performed. **(A)** In the RYGB procedure, the upper gastrointestinal tract is transected from near the gastric fundus and the jejunum to form a small gastric pouch. The stomach remnant and the duodenum and proximal jejunum are bypassed and attached to the mid-jejunum through a jejunal-jejunal anastomosis. Another gastro-jejunal anastomosis allows the connection of the gastric pouch and distal jejunum. **(B)** In VSG, most of the stomach is resected along the greater curvature while the remnant stomach forms a sleeve structure that greatly decreases the accommodative capacity and increases the chyme’s diversion rate.

**Figure 2:**
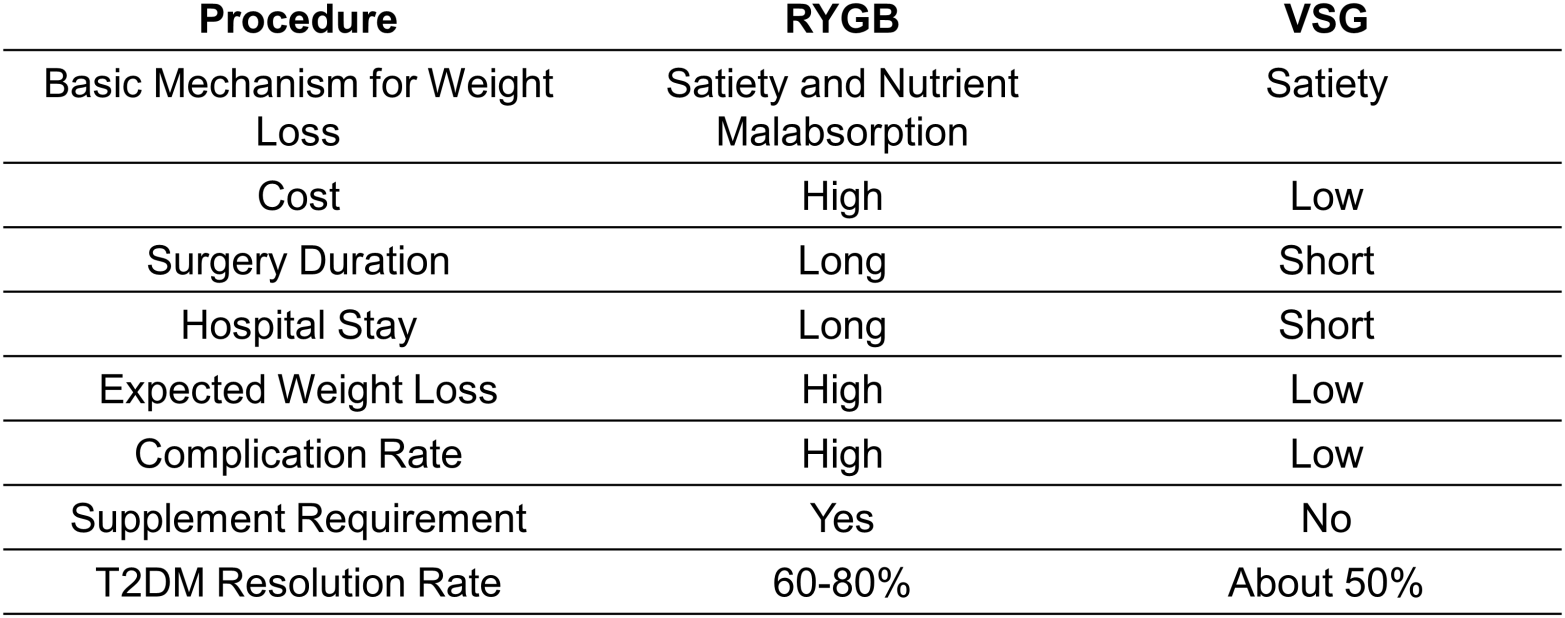
A comparison between two most commonly performed bariatric procedures.

The clinical benefits of BS are multi-faceted. At a population level, the average body weight loss expectation for patients receiving a bariatric procedure is expected to be over 60%, while approximately 80% of patients complicated by T2DM go into remission (9, 16). Two years following BS, about four-fifths of all diabetic patients still have sustained diabetes resolution (8, 9). The improvement in glucose tolerance can be sustained up to 5 years after surgery compared to simple lifestyle intervention or medications (17). Roux-en-Y gastric bypass (RYGB) and vertical sleeve gastrectomy (VSG) are the two most commonly performed bariatric procedures but exploit distinct surgical strategies (**Figure 1**; **Table 1**). The two approaches have slightly different, almost comparable clinical outcomes (**Figure 2**). While VSG is a cheaper, more straightforward, and less reconstructive approach, it has worse outcomes regarding body weight loss and T2DM remission rate (18, 19). Still, both procedures have higher expected weight loss than traditional vertical banded gastroplasty (VBG) and adjustable gastric banding (AGB) (20). Although BS has been proven safe and effective, the overall complication rate following BS was 10–18% (21). Short-term surgical complications, such as anastomotic gastric leaks (22), fistula tract formation (22), and small-bowel obstruction (SBO) (23), neurological disorders and muscle weakness, and nutritional deficiencies (24), can be dreaded in some patients. Long-term complications include gallstone disease (25) and bone loss (26). On the other hand, BS is not a ‘magic bullet’ to T2DM for all obese patients. At least 40% of patients seeking do not have adequate, long-lasting T2DM remission (27, 28). Even for patients with diabetes under control at the beginning, the remission does not seem to stay efficacious forever. About 20-30% of obese patients with an initial response experience relapse early or late within ten years (27, 28). The Swedish Obese Subjects (SOS) study demonstrated a 10-year relapse rate as high as 50% (29).

**Table 1:** Possible β-cell centered strategies that can supplement T2DM treatment for disease relapse after bariatric surgeries.

Strategy	Advantages	Disadvantages
Stem cell differentiation	• Standardized and unlimited supply of donor materials • No rejection issue	• Tumor risk • Incomplete understanding of induced differentiation *in vitro*
Stimulating β-cell proliferation	• Simple and controllable • No rejection issue	• Lack of agents with sufficient treatment effect
Reprogramming of non-β-cells	• Achievable through both *in vivo* and *in vitro* approaches • No rejection issue	• Failure of stable induced reprogramming of human cells
Xenogeneic pancreas transplantation	• Unlimited supply of donor organ • Promising β-cell function	• Strong rejection of donor cells
Allogeneic pancreas transplantation	• The full potential of β-cell function	• Shortage of donor organ • Rejection of donor cells

Despite the undisputable effect of BS on treating T2DM, there is only a limited understanding of the complicated mechanism behind the process, while even less is known about the mechanism that underlies the relapse of T2DM after surgery. The remission of T2DM was once considered a combined result of decreased food intake, nutrient absorption, and weight loss (30). However, this hypothesis was contradictory to the fact that glucose tolerance can be improved within days after the surgery, long before substantial weight change, indicating a glycemic control mechanism that is potentially independent of weight control (31–33).

In this review, we review the recent progress in the impact of BS on pancreatic β-cell and discuss novel therapeutics that have the potential to improve β-cell function and sustain the efficacy of surgery in the treatment of T2DM.

## Current recommendations for bariatric surgery

The recommendations for the patient population suitable for BS have remained almost unchanged over the last three decades (34). As recommended by most guidelines, BS should be considered for patients with a body mass index (BMI)≥40 kg/m^2^, BMI≥35kg/m^2^ with associated comorbidities that are expected to improve with weight loss, or BMI≥30–35 kg/m2 complicated with T2DM or arterial hypertension with poor control despite optimal medical therapy (35, 36). RYGB and VSG provide almost comparable effects regarding weight loss and T2DM treatment; thus, the choice of procedures should comprehensively consider institution experience, patient preference, and physician evaluation (6).

## The role of β-cell in the onset of obesity-related type 2 diabetes mellitus

In adults, pancreatic β-cell is a group of highly differentiated insulin-secreting cells (37). As an endogenous preventive mechanism, the β-cell responds to hyperglycemia with a compensatory increase in insulin secretion and cell mass expansion (38, 39). β-cell mass is not homogenous. The asynchronization of intra-islet cell differentiation, metabolic stress, and aging collectively lead to the overall heterogeneity of β-cell morphology and function (40). These cells are susceptible to autoimmune attack and distorted metabolic environments, characterized by progressive cell apoptosis under pathological conditions (41, 42). It is well established that excessive caloric or fat intake leads to insulin resistance and β-cell function impairment, as the central mechanism that underlies T2DM (43). In this model, a persistent hyperglycemic state poses so-called ‘glucotoxicity’ to pancreatic islets, which induces β-cell apoptosis and gradually results in the dysfunction of β-cell, secondary to the progressive loss of the β-cell population (44). Chronic insulin resistance has been shown to accelerate this process (40, 45, 46). Although constant cell renewing compensates for the reduction in β-cell mass before age 30 (47), the cell turnover rate gradually slows down, and finally, cell loss cannot be counterbalanced by new cell replacement anymore beyond this age (48).

Systemic low-grade tissue inflammation is a hallmark of obesity (49). Widespread tissue immune cell infiltration and release of proinflammatory factors collectively promote insulitis and β-cell loss (50). Obesity also induces the proliferation of islet-resident M1 macrophages, which account for local inflammation and impairs β-cell function (51, 52). Similarly, enhanced TNF-α and CCL2 expression were correlated with hampered glucose-stimulated insulin secretion (GSIS) (53).

Whether the overall islet degeneration is because of β-cell death or loss of function once remained debated (54). Talchai et al. proved that, during the pathogenesis of T2DM, matured β-cells lost their identity and reverted to progenitor-like cells expressing naïve markers, including L-Myc, Nanog, and Oct4 (55). By morphometric quantification, an average of 30% of β-cells underwent dedifferentiation in donor islets from the T2DM group, while less than 10% in nondiabetic controls (56). The dedifferentiation was reversible. Evidence showed that a fraction of dedifferentiated cells could again transdifferentiate into glucagon-secreting cells that resemble α-cells (55). Insulin therapy can induce the dedifferentiated cell to re-differentiate to mature neurogenin3-negative, insulin-positive β-cells (45), possibly the primary mechanism for that intensive insulin therapy improved β-cell function and resulted in prolonged glycemic remissions (57).

## Bariatric surgery promotes T2DM remission in a weight-independent manner

The role of BS in the remission of T2DM remains partially understood. Many plausible mechanisms, which are not necessarily exclusive to each other, have been proposed in recent years (58). Traditional views held that food intake restriction, nutrient malabsorption, and subsequent weight loss caused by altered gastrointestinal (GI) anatomy is the central mechanism for T2DM resolution shared by various bariatric procedures (59). However, several lines of evidence indicated that the improved glycemic control is independent of weight loss and calorie intake, at least partially. First, it was observed that the remission rate of T2DM in leaner individuals is similar to that in severely obese patients (60), indicating that the correlation between glucose tolerance control and weight change was disproportional. Second, the improvement of glucose homeostasis, insulin secretion, and insulin sensitivity usually occurs days after the surgery, long before significant weight loss, and regardless of specific bariatric procedures (19, 61). Third, such rapid changes only exist in RYGB or VSG that require digestive tract reconstruction but not in restrictive surgical techniques such as vertical banded gastroplasty (VBG) and laparoscopic adjustable gastric banding (LAGB), which promote weight loss solely by decreasing nutrient intake (62). All this evidence provided evidence of the necessity to divide the glucose-lowering effect of BS into the early effect and the late effect. While the late effect, or weight loss, plays a vital role in the durable remission of T2DM. Researchers realized that the early effect was potentially a combined result of restriction of gastric volume, lowered nutrient absorption, increased gastric emptying rate, postprandial synthesis of secretin, altered GI hormone secretion release pattern, and sensitized hepatic insulin response regardless of specific procedures (31, 63–65). These tend to be immediate effects of GI reconstruction, after which insulin sensitivity and glucose tolerance are improved prior to significant weight change (66, 67). As the primary causative factor of T2DM in the case of obesity, the impaired β-cell function is largely alleviated after BS, and the alteration has been gradually considered central to the remission of T2DM (68). In the following sections, we will focus on the functional and physiological change of pancreatic β-cells and the driving factors that may govern that change after RYGB and VSG.

## Bariatric surgery promotes T2DM remission by modulating β-cell function

### The role of β-cell in RYGB

**Figure 3:**
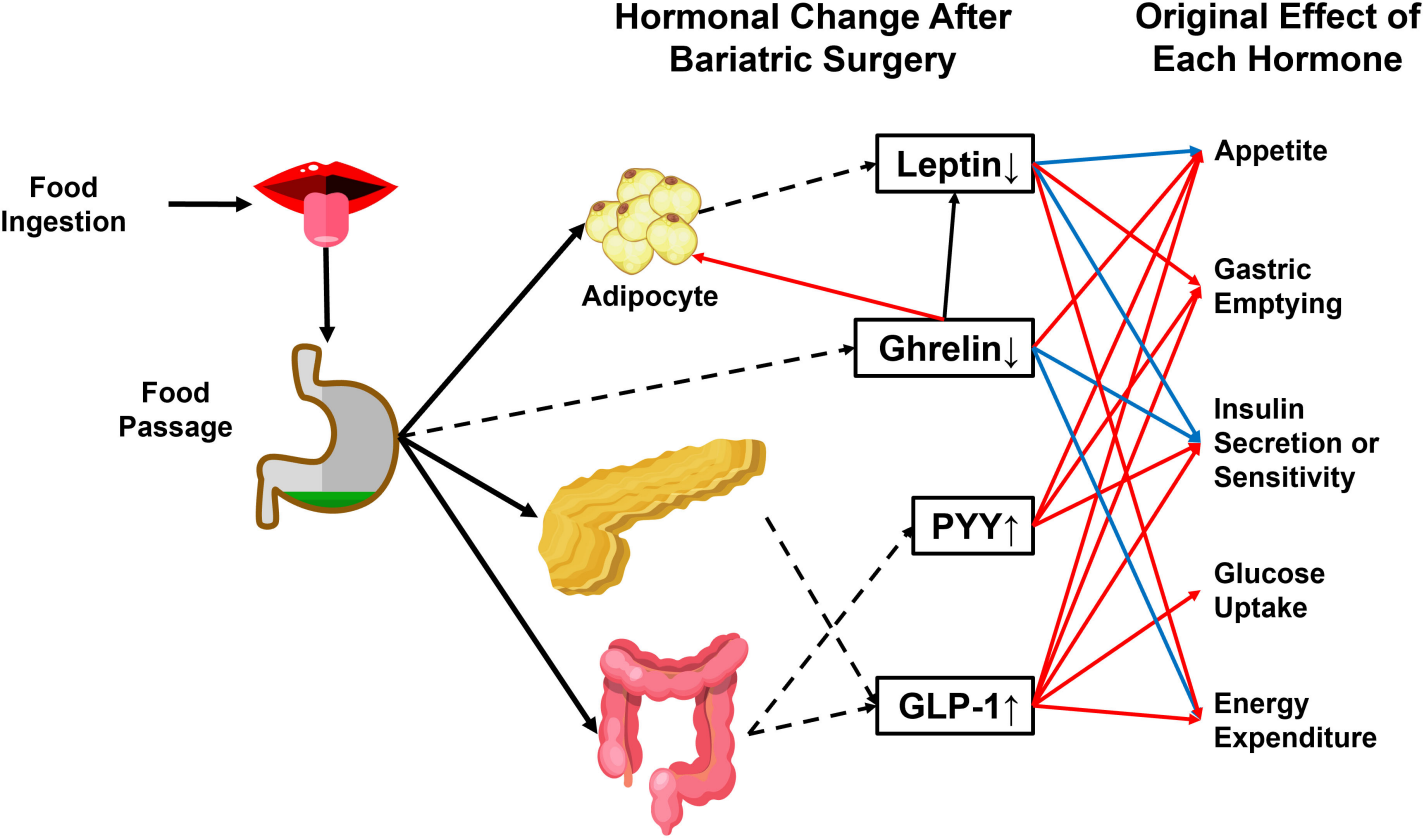
Physiological effects of crucial hormones that are altered after bariatric surgery. GLP-1 and PYY are gut-secreted hormones with anti-obesity and antidiabetic actions. The passage of food can stimulate both hormones through the gastrointestinal tract. Ghrelin and leptin are reduced after bariatric surgery. Ghrelin induces energy storage in the form of fat by promoting adipose tissue deposition and fatty acid oxidation, reducing energy expenditure, and blocking insulin secretion. Leptin has an antagonistic effect on ghrelin. PYY, Peptide YY. GLP-1, glucagon-like peptide 1. Dashed arrowhead, hormone secretion. Blue arrowhead, inhibitory effect. Red arrowhead, stimulatory effect.

The RYGB procedure involves creating a small stomach pouch connecting to the rerouted small intestine (Roux limb) (69). Since the upper intestine senses nutrient passage and activates a gut-brain-liver axis to maintain glycemia under physiological conditions (70), the exclusion of duodenum from the main gastrointestinal tract (GI tract) breaks the balance by leading to the delivery of a large amount of insufficiently digested food to the lower digestive tract. It induces the secretion of strong gut hypoglycemic hormones, mainly glucagon-like peptide (GLP-1) and peptide YY (PYY) (71) (**Figure 3**).

For a long time, it was observed that diabetes remission was affected by the time of diabetes duration, poor preoperative glycemic control, and preoperative insulin dosage. People inferred that perhaps less deterioration in β-cell mass or function before surgery might maximize the effect of the surgery-induced pattern change of gut peptides that glucose balance. As verified in the male Zucker Diabetic Fatty (ZDF) rat model, the RYGB procedure efficiently restored pancreatic islet function and improved glucose tolerance in males (72). Increased insulin sensitivity and enhanced insulin secretion improved glucose metabolism (72). Histopathologically, pancreas hyperplasia (73) and β-cell regeneration (74) were observed shortly after RYGB. Compared to the sham surgery group, there was a 60% increase in β-cell mass in the RYGB rat islets three months post-surgery, possibly resulting from islet neogenesis (73). Preservation of β-cell mass was also observed in Goto-Kakizaki (GK) rats (75). Tracking of mature β-cell markers indicated a decreased ratio of dedifferentiated cells (76) and apoptotic β-cell (77). However, considering the pronounced differences in gastrointestinal tract structure, nutrient composition, and endocrine pattern, results in rodent models may not be easily translated into a human.

In porcine models, more significant pancreatic islet number, mass size, and improved β-cell function were observed receiving RYGB surgery compared to the diet control group (78). However, other evidence exists to support that β-cell mass remains unchanged after BS (32, 79, 80). Some groups argue that the elevated expression of cell markers such as insulin, Pdx-1, Maf A, Pcsk1, and Glut2 are outcomes of improved glycemia rather than drivers (31). As proof of the argument, the ex vivo β-cell function was not improved (32).

In real-world T2DM patient cohorts, altered insulin secretion and sensitivity can independently or jointly improve glucose homeostasis after RYGB, as measured by various available techniques (81–84). As mentioned previously, the glucose-lowering effect can be divided into early- and late-stage. In a prospective study, the oral glucose tolerance test (OGTT) was measured immediately and one year after RYGB in 119 morbidly obese participants, indicating sustained late-stage islet function improvement (82). The calculated disposition index (DI) and proinsulin-to-insulin (PI/I) ratio of the surgery group were significantly higher than the lifestyle intervention group (82). However, the early-stage data was lacking. To present a longitudinal comparison of early-stage and late-stage β-cell function, Nannipieri et al. measured glycosylated hemoglobin and OGTT after 45 days and one year after surgery and mathematically correlated blood glucose, insulin, and C-peptide concentrations (83). They observed sustained insulin sensitivity improvement regardless of the existence of T2DM, while the background β-cell function before the surgery was a significant determinant of T2DM remission (83). The enhanced insulin response was durable as measured during a two-year observation (81, 84).

The exact mechanism underlies the alteration of β-cell physiology and improved glucose homeostasis after gastric bypass is mainly elusive. The rapid amelioration of islet function has multiple potential explanations. In Nannipieri’s experimental design, the designated nutrient uptake after RYGB was roughly 800 kcal (83), while the effect of calorie restriction or fasting on plasma glucose equilibrium and insulin sensitivity has been well documented (66). It is of equal importance that the rapid post-surgery increase in incretin hormones or bile acids can specifically potentiate β-cell function (85). Altered gut hormone signaling for the reconstructed GI tract was considered a leading cause of insulin secretion and increased blood glucose uptake (86). GLP1 and gastric inhibitory polypeptide (GIP) are two major GI antidiabetic hormones that activate insulin secretion on oral ingestion of food, and each exerts its effect by binding to its receptor (75, 87). The impaired GLP-1 and GIP secretion and function in obese patients were partially restored after gastric bypass surgery or VSG, compared to those who received a low-calorie diet with equivalent weight loss (75, 88, 89). In an obesity cohort (BMI≥35), the post-surgery usage of Ex-9 (a specific GLP-1R antagonist) completely reversed improvements in β-cell glucose sensitivity and glucose tolerance, implicating the critical role of activated GLP-1 response in this process (90). In rare cases, the hyperactivated GLP-1 secretion leads to hyperinsulinemic hypoglycemia, a life-threatening post-RYGB complication that usually arises years after the surgery (91), which highlights the role of the proximal small intestine. In the GK rat model, Ramracheya et al. demonstrated that glucagon secretion was restored after RYGB, while this effect was dependent on a gut hormone, PYY, but not GLP-1 (92).

It is likely that distinct mechanisms account for late-stage post-surgery islet function improvement. Chronic glucose toxicity is one of the leading causes of β-cell degeneration during T2DM development and the fact that β-cell can self-renewal, the removal of glucose toxicity at the early stage caused by other mechanisms at least partially accounts for the prolonged individual glucose-sensing ability and insulin secretion (93). A further hypothesis is that the abatement of insulin resistance decreases the workload of β-cells. Improvement of homeostasis model assessment of β-cell function (HOMA-β) was observed (51).

A later study demonstrated that the Roux limb’s morphological and metabolic remodeling adaptation, characterized by intestinal mucosa hyperplasia and hypertrophy, is responsible for improved glucose homeostasis (94). Enhanced GLUT-1–mediated glucose transportation and utilization can be observed under rat abdominal positron emission tomography-computed tomography (PET-CT), which contributes to improved whole-body glucose disposal (94).

### The role of β-cell in VSG

As a procedure gaining popularity, VSG has surpassed RYGB to become the most performed BS (95). The procedure involves removing 80% of the stomach by transecting along the greater curvature while preserving the intestinal anatomy’s original integrity (96). VSG can help obese patients to achieve weight loss and T2DM remission almost comparable to RYGB, with more straightforward modifications to the GI tract, fewer surgical risks, and reduced postoperative complications (95).

In obese patients, glucose control is quickly restored after VSG, as indicated by apparent reductions in both prandial and fasting glucose levels after the surgery and improvement of insulin resistance (32). VSG promotes β-cell mass increase and cell function restoration as short-term responses (32, 97–100). Furthermore, histopathological sections of the pancreas displayed a decrease in pancreatic lipid droplet infiltration (32), restoration of islet integrity (99), and β-cell expansion (97) shortly after VSG. Using a positron emission tomography (PET) combing ligand-receptor marking technique, Inabnet et al. were able to demonstrate an increase of Vesicular monoamine transporter type 2 (VMAT2) positive β-cell mass (100). Grong et al. arrived at a similar conclusion and applied a three-dimensional optical projection tomography that can precisely visualize insulin-secreting cells by antibody staining (98). VSG rats had a significantly higher beta-cell density than duodenojejunostomy rats or sham (animals that underwent a false abdominal surgery without changing the GI anatomy) rats (98).

Whether increased insulin secretion or improved insulin sensitivity is the leading cause of glucose homeostasis after VSG remains inconclusive. Abu-Gazala et al. and other groups observed that the operation does not directly affect insulin secretion, while the improved hepatic insulin sensitivity after the surgery played a vital role in the remission of T2DM (32). Other groups hold the view that VSG promoted GSIS (99, 101).

One of the most dramatic post-surgery hormonal changes shared by various bariatric procedures, including VSG, is an over 10-fold increase in the postprandial secretion of GLP-1, a well-established potent stimulator of GSIS (16). There are two distinct sources for GLP-1 elevation after BS. First, the increased influx of insufficiently absorbed nutrients stimulates the secretion of GLP-1 by enteroendocrine L cells in the distal gastrointestinal tract in response to a meal (102). The other source lies within the pancreatic islet. Garibay et al. exploited the β-cell-specific inducible GLP-1R knockout murine model to investigate the role of intra-islet GLP-1R signaling change after VSG. Their result demonstrated that increased GLP-1 in the systemic circulation activated the GLP-1 secretion by α-cell in a β-cell GLP-1R-dependent manner, possibly forming an intra-islet positive feedback loop that promotes insulin secretion (103). However, the exact association between the two GLP-1 secretion pathways remains unclear.

Although the surgery-induced activation of the GLP-1 pathway plays a critical role in GSIS and T2DM remission, it may not be unique. However, patients with diabetes with VSG develop only modest glucose intolerance when treated with the GLP-1R antagonist exendin-[9], similar to a non-operated control group. The lack of disproportionate glucose intolerance during GLP-1R blockade in surgical subjects speaks against increased GLP-1 making a significant contribution to the metabolic improvement after surgery.

In *db/db* mice (a murine model that simulates T2D status related to morbid obesity), VSG reduced glycemia independent of weight loss (32). Single-cell transcriptomic analysis of intra-islet cells acquired from obese mice reveals that in β-cells, the ER-associated protein degradation pathway (ERAD) was activated (97). In the mice that received VSG, the endoplasmic reticulum stress was mitigated due to the augmentation of unfolded protein response. Moreover, the surgery-induced transcription change was exclusive to β-cell rather than other endocrine cells in the islet (97). While in the sham-controlled group, the intra-islet cells retained features of compromised β-cell identity and function and extreme dedifferentiation (97). Using a similar transcriptomic analysis combining pathway enrichment, Douros et al. found an evident activation of calcium signaling for the VSG group mice (101). They hypothesized and verified that increased intra-islet Ca2+ oscillation amplitude in the islet without changing the insulin secretory capacity (101). This alteration fundamentally re-sensitized the islet to glucose concentration, and ex vivo measurement indicated a persistent effect (101).

Since most current studies of the impact of VSG or RYGB on T2DM use mouse models, we should hold a critical understanding of the results by emphasizing a few points. Based on current evidence, although RYGB and VSG lead to comparable weight loss and β-cell function improvement in obese patients, RYGB in mice provides more profound and sustained body weight loss than VSG. The gap between human and animal models has been disputed and recursively highlighted in all biomedical research. Second, it is essential to remember that although the therapeutic effect of bariatric procedures can be compared using unified clinical indicators such as weight loss and insulin concentration, the mechanisms underpinning various procedures can be strikingly different. Even for the same procedure, for example, VSG, the impact of diverse, well-established surgical mouse models and different techniques may have been underestimated (104). As pointed out by Myronovych et al., each of the cohorts of mice used in various studies is of various ages, differing diets, and different lengths of exposure to the diet. The mice’s starting weights and body fats also varied considerably from one cohort to another (105). A critical judgment of the effect of BS conferred from animal experiments cannot be overemphasized.

## Add-on strategies to promote β-cell regeneration

As mentioned previously, the regenerative ability of pancreatic islets is limited, and they gradually experience cell loss during individual development and growth (106). Although BS has the potential to reverse obesity-induced β-cell dedifferentiation and partially restore cell function, the unavoidable cell degeneration may underlie the postoperative relapse of diabetes and reemergence of other obesity-associated comorbidities (27, 28). Untangling the β-cell pathophysiology during diabetes pathogenesis and BS has shed new light on the development of add-on therapy to mitigate or prevent disease relapse.

The initial success of stem cell therapy (107), hormones therapy, and cytokine therapy (108) in preclinical animal models remind us that the islet regenerative strategy may be a powerful method for adjuvant management of obesity-related T2DM and can be achieved at multiple levels (106). Endogenously, halting β-cell dedifferentiation to maximally preserve β-cell mass and function at an early stage of the disease is fundamental. This goal can be achieved by insulin treatment (57) or diet control (107, 109–111). In db/db mice, simple calory restriction can prevent and possibly reverse β-cell dedifferentiation, as certified by decreased expression of progenitor marker Aldh1a3 and enhanced β-cell markers such as MafA, NeuroD1, and Foxo1 (111). On the one hand, the calory-restricting diet plan overcomes high-fat diet-induced changes and recovers islet calcium oscillation coordination necessary for insulin secretion (112). Conversely, the fasting-mimicking condition upregulated Ngn3 expression, thus promoting β-cell proliferation and regeneration (113). The application of insulin and other antidiabetic medications permits temporary reduction of systemic hyperglycemia promoted redifferentiation of dedifferentiated β-cells (114–118).

Another approach is to replenish the target cells *via* transplantation of β-cells from donor pancreata or cells derived from human pluripotent stem cells (hPSCs) (106, 119). A shortage of donor organs has largely impeded the clinical feasibility of the former choice; thus, stem cell-based transplantable β-cells therapy has been spotlighted recently (120). The development of β-cells followed a strict pattern regulated by the sequential expression of specific transcription factors (121). In mouse embryos, multipotent pancreatic progenitor cells co-express the critical transcription factors Sox17 and Pdx1 (122). These progenitors then converted epithelial cells with a bipolar destiny: pancreatic duct cells or Ngn3+ precursors that later give rise to all the endocrine in the pancreatic islet, including α cells and β-cells (123). As a further step toward the *in vitro* cell culture of fully functional β cells, Nair et al. first stressed the importance of cell re-clustering after β cell induction from hPSCs to resemble their endogenous counterparts maximally (107). These insights are now exploited to recapitulate aspects of islet formation *in vivo* and formulate novel regenerative tactics by differentiating pluripotent stem cells or reprogramming non-β-cells into transplantable β-cells that may complement BS in the management of T2DM.

## Discussion

Numerous high-quality clinical trials have verified the clinical benefits of BS in treating T2DM, making it currently the most effective treatment to control obesity-related T2DM. Nevertheless, heated debate existed on its actual necessity and safety. Although an increasing number of obese patients seek BS, the proportion is lower than 1% of the vast obesity population (124). First, drawbacks exist. The overall mortality rate of BS is 0.5-2%, according to studies based on ample research populations (125–127). The overall complication rate following BS was 10–18% (21), including short-term surgical and long-term nutritional complications.

On the other hand, BS seems not to be a cure for all obese patients. About 40% of patients do not have sustained T2DM remission, and 20-30% of patients with an initial response experience relapse early or late within ten years (27, 28). The SOS project reported a 10-year relapse rate as high as 50% (29).

For one thing, the accurate screening of obese patients that benefit most from the surgery is critical to further recognition and application of BS. Predicting tools based on machine learning methods have been utilized for diabetes remission outcomes after BS. Pedersen et al. developed a heuristic method and concluded that baseline HbA1c and insulin levels, doses of antidiabetic agents such as insulin and insulin-sensitizing medication, and several single nucleotide polymorphisms (SNPs) were informative variables that were related to the response of the surgery (128). Note that all the SNPs had a reported role in insulin secretion, insulin sensitivity, or obesity. Other scoring systems, such as ABCD, IMS, and DiaRem, have also been proposed for short- or long-term diabetes remission (129, 130). However, these scores were developed according to specific bariatric procedures, and none has sufficient predicting power for all patients (131).

While for another, the question needs to be answered why diabetes cannot be controlled after surgery or relapse in certain patients; before that, it is of prime interest to fully understand why BS is effective in the first place. It is indisputable that the mechanism behind surgery-induced T2DM remission is muti-dimensional. Though weight loss has been proven to be a crucial contributor to glycemic control in various bariatric procedures, an increasing body of evidence supports a distinct, weight-independent glucose-lowering mechanism.

As summarized in this review, chronic islet inflammation and glucotoxicity among obese patients progressively impair β-cell function by inducing cell apoptosis and dedifferentiation, while the recovery of β-cell morphology, cell mass, and GSIS after RYGB and VSG seem to be a major cause of rapid improvement of glucose homeostasis. Increased secretion of certain GI hormones accelerates islet function restoration. More research focuses are warranted in the future. First, most current studies with animal models and patients focus on the short-term alteration of islet function, while a longitudinal tracking of the β-cell function after the surgery is lacking. Second, a valid comparison of β-cell function between surgery responders and nonresponders may indicate whether β-cell function improvement is a central indicator of surgery efficacy. Third, the monitoring of β-cell function in initial responders that experiences a late T2DM relapse can help to provide us with insights on novel therapeutics to improve the surgical outcome. Last but not least, Jiménez et al. observed that patients experiencing a later relapse had poorer baseline β-cell reserved function before the surgery, as reflected by dependence on heavy insulin or multiple glucose-lowering agents (98, 99). Correlating the baseline islet function of obese patients and long-term diabetes remission status is critical for choosing the right patient to obtain the most significant benefit from surgery and discriminating the population prone to diabetes relapse that may need additional disease-modifying treatments.

There are no solid and unified recommendations for surgery-refractory obesity or postoperative recurrence due to a lack of high-quality clinical research. Although some pharmacological interventions such as anti-inflammatory and GLP-1 receptor agonists can temporarily improve islet function (132, 133), there is no consistent evidence to support that preoperative or postoperative addition of these drugs and surgery can increase the response rate or lower the diabetes recurrence rate of BS. To elucidate the islet pathophysiology is of critical significance to the understanding of surgical failures. On the other hand, although our current understanding of how the pancreas develops during embryogenesis is scarce, attempts are being made to formulate regenerative strategies that center on induced differentiation of human pluripotent stem cells (hESCs) or reprogramming of non-β-cells. While promising preclinical studies and clinical trials of hESC-induced islet cell regeneration are ongoing, breakthroughs in this direction may hopefully provide us with a radically new strategy to manage obesity-related T2DM and increase the efficacy of BS.

## Ethics statement

The authors certify that they have obtained the participant consent forms. In the forms, participants have given their consent for their images and other clinical information to be reported in the journal. The participants understand that their names and initials will not be published, and due efforts will be made to conceal their identity.

## Author contributions

TL was responsible for the literature searching and writing. RR and XZ were responsible for the manuscript revision and figure production, and QX was responsible for the supervision and manuscript revision. All authors contributed to the article and approved the submitted version.
